# Heparin-Induced Thrombocytopenia After Mitral Valve Replacement

**DOI:** 10.31486/toj.20.0007

**Published:** 2021

**Authors:** Carmen Tugulan, Donald D. Chang, Michael J. Bates

**Affiliations:** ^1^ Section of Cardiothoracic Surgery, Department of Surgery, Ochsner Clinic Foundation, New Orleans, LA; ^2^ The University of Queensland Faculty of Medicine, Ochsner Clinical School, New Orleans, LA

**Keywords:** *Cardiac surgical procedures*, *heparin*, *mitral valve*, *thoracic surgery*, *thrombocytopenia*, *thrombocytopenia–heparin-induced*

## Abstract

**Background:** Heparin-induced thrombocytopenia (HIT) is a rare autoimmune reaction that involves a decrease in platelet count following heparin exposure and can be associated with life-threatening thrombosis. Because of their prolonged heparin exposure, patients undergoing cardiac surgery are at risk of HIT, with an incidence of 0.1% to 3%.

**Case Report:** A 65-year-old male with severe mitral regurgitation and preoperative ejection fraction of 20% to 25% underwent mitral valve bioprosthetic replacement with coronary artery bypass graft surgery. Heparin anticoagulation was started on postoperative day (POD) 1. Respiratory failure resulted in prolonged mechanical ventilation and heparinization without the ability to initiate warfarin. While the patient was on heparin, his platelet count declined on POD 2 and then steadily increased to above the preoperative level on POD 7. On POD 10, the patient's platelet count dramatically decreased, and on POD 13 he developed acute common femoral artery occlusion necessitating embolectomy. Intraoperative transesophageal echocardiography revealed heavy thrombus burden across the mitral bioprosthesis. HIT was confirmed with a positive heparin-induced platelet antibody and serotonin release assay. Heparin was stopped and argatroban initiated. The patient underwent reoperative bioprosthetic mitral valve replacement on POD 18 using bivalirudin intraoperatively. Despite resolution of HIT, the patient developed sepsis and died on POD 59.

**Conclusion:** The diagnosis of HIT is challenging in patients who undergo cardiopulmonary bypass. Platelet counts often decrease 40% to 60% during the first 72 hours postoperatively, and the frequency of nonspecific anti–platelet factor 4/heparin antibody formation is high. These findings can mask early signs of HIT and delay diagnosis.

## INTRODUCTION

Heparin-induced thrombocytopenia (HIT) is a rare autoimmune reaction that involves a decrease in platelet count following heparin exposure and can be associated with life-threatening thrombosis (heparin-induced thrombocytopenia and thrombosis). First described in 1958, HIT is widely considered to be one of the most severe postoperative complications for patients treated with heparin^1-4^ and has 2 types. HIT I is a relatively benign diagnosis with no clinical symptoms and is characterized by a transient drop in platelet count that self-resolves with no antibody-mediated sequelae. In contrast, HIT II presents with a platelet count decrease associated with significant clinical symptoms, autoimmune antibody-mediated damage, and possible thromboembolic sequelae. Risk factors for HIT II include female sex, age >65 years, prolonged heparin exposure, use of unfractionated heparin (UFH), and surgical procedures.^5-7^ Both clinical findings and laboratory results are necessary for diagnosing HIT because neither is sufficient alone for making the diagnosis. Management includes immediate cessation of heparin and initiation of therapeutic anticoagulation.

Patients undergoing cardiac surgery often have many of the risk factors for HIT II, in particular extended exposure to UFH at a high dose and use of cardiopulmonary bypass (CPB). Patients undergoing cardiac surgery have incidence rates of HIT as high as 3%, a sharp contrast to the reported 0.1% to 0.3% rate in the general patient population.^8-10^ Early recognition and treatment are further obscured by the fact that thrombocytopenia routinely occurs after cardiac surgery. The severity of HIT II in patients undergoing cardiac surgery was underscored by a 1992 study reporting a post cardiac surgery mortality rate up to 28%.^11^ However, the paradigm of HIT II as a devasting and high-mortality complication is shifting; a 2016 study has suggested that early detection and intervention can lead to significantly reduced mortality rates of approximately 1%.^12^

We report the postoperative course and management of a patient who acquired HIT II after cardiac surgery.

## CASE REPORT

A 65-year-old male was evaluated for mitral valve bioprosthetic replacement and coronary artery bypass graft (CABG) surgery in a setting of multivessel coronary artery disease and severe mitral regurgitation. His medical history was significant for ischemic cardiomyopathy, prior percutaneous intervention, hypertension, and hyperlipidemia.

**Figure 1.**
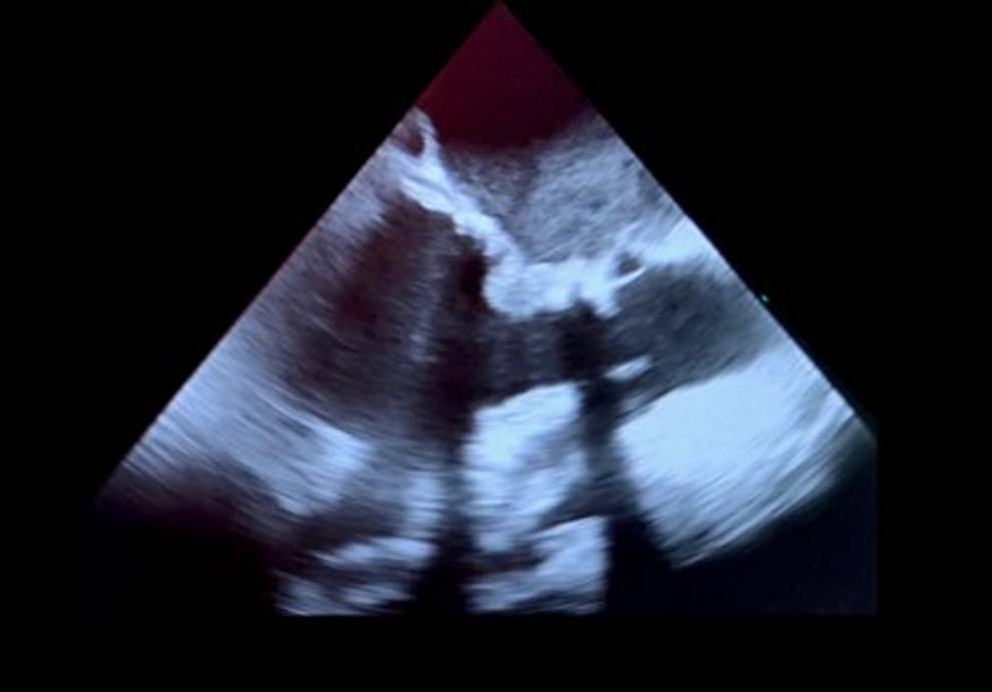
Preoperative ultrasound transesophageal echo-cardiography shows the thickened mitral valve obstructing cardiac vascular flow.

Cardiac catherization revealed complete occlusion of the left circumflex artery, 90% occlusion of the right coronary artery, and 95% occlusion of the diagonal branch of the left anterior descending artery. The patient received 11,750 U of UFH without complication. Two-dimensional echocardiography revealed 0.3 cm^2^ effective regurgitant orifice area across the mitral valve, 60 mL regurgitant volume, and regurgitant fraction of 54% consistent with severe mitral regurgitation (Figure 1). The patient also had a severely depressed preoperative ejection fraction of 20% to 25% and severe left ventricular enlargement. Preoperative platelet count was 164 K/μL.

The patient underwent CABG and mitral valve replacement utilizing 52,000 U of UFH for CPB. Cross-clamp time was 67 minutes, and the patient's immediate postoperative platelet count was 108 K/μL. The patient remained intubated and sedated postoperatively because he did not tolerate spontaneous breathing trials. Heparin infusion of 500 U/h was started on postoperative day (POD) 1. Postoperative prolonged mechanical ventilation and small bowel ileus necessitated maintenance of nothing-by-mouth status, thus delaying initiation of oral anticoagulation with warfarin. The patient's platelet count declined sharply on POD 2 (66 K/μL) before trending upward on POD 3 (82 K/μL) and POD 4 (97 K/μL). The patient was extubated on POD 6. Platelet counts continued trending upward and were stable by POD 7 (242 K/μL) and POD 8 (230 K/μL). On POD 9, the patient developed atrial fibrillation with rapid ventricular response and underwent successful electrical cardioversion.

On POD 10, the patient's platelet count acutely decreased to <100 K/μL from 149 K/μL the previous day, and the patient was reintubated because of respiratory failure. His platelet counts continued to trend downward and reached a nadir of 70 K/μL on POD 13 when physical examination noted the absence of left lower extremity pulses. Arterial duplex ultrasound revealed acute common femoral artery occlusion, and emergent embolectomy was performed. Heparin was discontinued, and argatroban 2.5 μg/kg/min was initiated. Intraoperative transesophageal echocardiography revealed heavy thrombus burden across the mitral bioprosthesis, causing severe mitral stenosis. Repeat echocardiography on POD 14 showed persistence of thrombus with a mean gradient of 20 mmHg across the mitral valve. Detection of heparin-induced antiplatelet antibody from enzyme immunoassay and positive serotonin release assay (SRA) confirmed the diagnosis of HIT.

**Figure 2.**
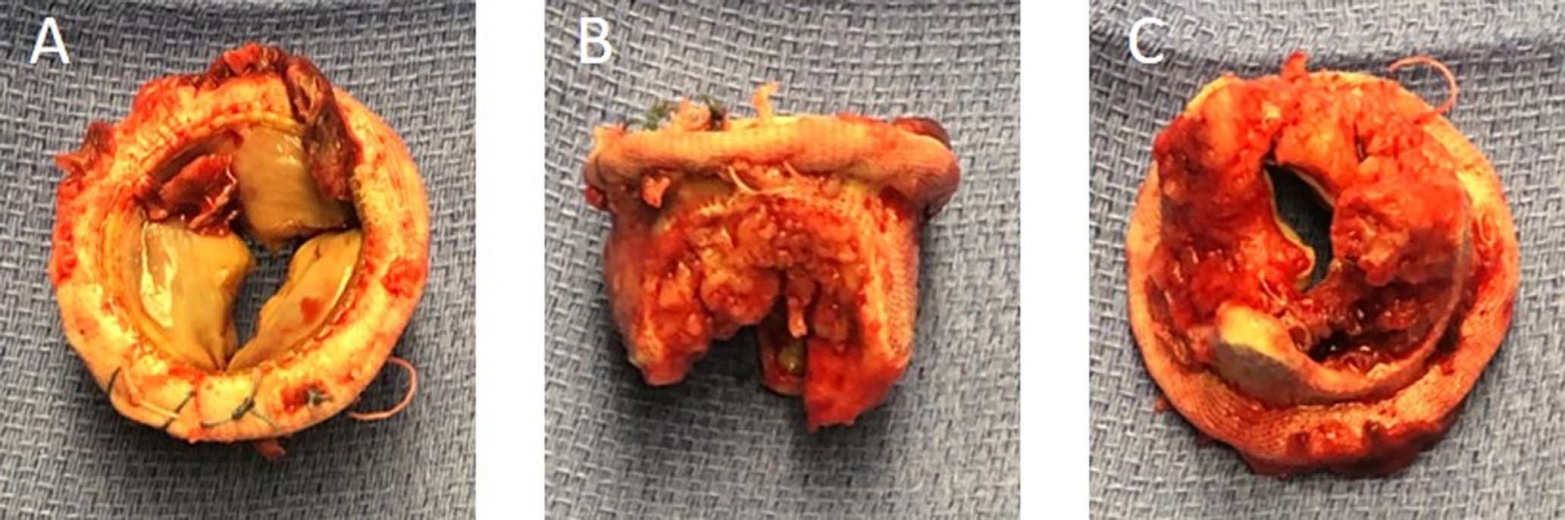
(A) Superior, (B) sagittal, and (C) inferior views of the resected thrombosed mitral valve show the thrombotic debris on the thickened leaflet walls.

Because of respiratory failure and severely thrombosed mitral valve, the patient underwent reoperative bioprosthetic mitral valve replacement on POD 18, with bivalirudin anticoagulation used continuously throughout the procedure. A loading dose of 120 mg bivalirudin was delivered upon initiating CPB to achieve an activated clot time 2 to 2.5 times baseline, followed by continuous infusion dosed at 2.5 mg/kg/h. The procedural approach was transseptal, and access was gained through the previous incision. Upon visualization, the mitral valve prosthetic showed dense adhesions and rigid white clots on both sides of the leaflets (Figure 2). The adherent clots effectively occluded the mitral valve orifice. All debris was debrided and pledgeted sutures were removed, followed by retrieval of the prosthetic valve. The posterior leaflet was almost completely resected and copious irrigation applied. No thrombus formation in either the operative field or the CPB circuit was encountered during the operation. A 31-mm Mosaic bioprosthetic valve (Medtronic) was implanted. Intraoperative echocardiography showed improved mitral valve function with a 1- to 2-mmHg gradient. Postoperatively, the patient's platelet counts were stable and remained >150 K/μL on argatroban 1 μg/kg/min.

Despite resolution of HIT, the patient's renal function worsened postoperatively, and he developed acute kidney injury, believed to be secondary to ischemic acute tubular necrosis from multiple hypotensive episodes intraoperatively (systolic blood pressures in the 70s and 80s mmHg) and requiring pressor support. Respiratory function also deteriorated postoperatively, and despite extubation on POD 6 after his second surgery, the patient was reintubated on POD 12. The patient could not be weaned from the ventilator and had a percutaneous tracheostomy tube inserted on POD 21. The patient developed sepsis on POD 30 with multiorgan system failure. Because of the patient's respiratory and renal failure, unremitting sepsis, and low chance of meaningful recovery, the family withdrew care on POD 41 after the second surgery, approximately 2 months (POD 59) from the primary mitral valve replacement procedure. The patient died on POD 59.

## DISCUSSION

HIT II is a life-threatening complication with an incidence rate of 0.1% to 0.3% in the general patient population, a 50% increase in mortality compared to patients who do not develop HIT, and mortality rates as high as 28% among patients undergoing cardiac surgery.^9-11^ HIT develops because of the production of autoantibodies against the platelet factor 4/heparin dimer complex that cause abnormal platelet activation and stimulation of the coagulation pathway.^13^

The increased presence of HIT in the cardiac surgery population can be attributed to 2 risk factors that the majority of these patients endorse: prolonged exposure to UFH and use of CPB. Signs and symptoms typically present 5 to 10 days postoperatively. Our patient was on CPB and had prolonged UFH exposure during his CABG and mitral valve replacement surgery, significantly increasing his risk of developing HIT.

Adverse outcomes from HIT include stroke, acute renal failure, and amputation secondary to thrombotic events. While venous instead of arterial thrombotic events are more likely to occur in general among patients with HIT, the majority of thrombotic events in patients undergoing cardiac surgery are arterial and have been observed to be 8.5 times more common compared to venous thromboses.^4,14^ Despite severe thrombocytopenia, bleeding and petechiae are rarely seen in patients with HIT.^15^ Our patient developed an arterial embolus in the left common femoral artery on POD 13 and required an emergent embolectomy, underscoring the life- and limb-threatening adverse events associated with HIT.

Definitive diagnosis of HIT is based on 2 laboratory tests: an immunoassay to detect anti–platelet factor 4/heparin antibody in the patient's serum and a functional assay to assess whether HIT antibodies from patient's serum can activate platelets, indicating abnormal stimulation of the coagulation pathway. The 2 main types of assays are the SRA, which measures serotonin release from platelets in presence of patient serum, and the heparin-induced platelet activation, which measures platelet aggregation in the presence of patient serum. Our patient tested positive for anti–platelet factor 4/heparin antibody and had a positive SRA, but in practice, these tests take several days to perform, so best practice is to make a presumptive diagnosis of HIT using clinical findings and probability scores.

The Warkentin 4Ts scoring system is one of the most widely used and clinically validated scoring systems for determining the pretest probability of HIT.^16,17^ The Warkentin 4Ts scoring system consists of 4 thrombocytopenia-related criteria: (1) severity, (2) timing, (3) presence or absence of thrombotic event, and (4) likelihood of other causes. Each criterion is assigned 0 to 2 points, and a score of 6 to 8 points indicates a high probability of HIT, while a score of 0 to 3 indicates low probability. A prospective study using the 4Ts criteria for HIT found that 11.4% of patients with intermediate scores and 34% of patients with high probability scores had clinically significant HIT antibodies.^16^ Our patient had a Warkentin 4Ts score of 4 prior to his thrombotic event and a score of 8 after, correlating to intermediate and high probabilities, respectively. Other probability scoring systems include the Lillo-Le Louët model, which is intended for use exclusively after CPB, and the HIT Expert Probability Score, which was developed from expert opinion.^18,19^

Among patients undergoing cardiac surgery, a decrease of >50% platelets occurring 5 to 10 days postoperatively can be highly suggestive of HIT.^18,20,21^ Our patient experienced a 69.6% drop in platelet counts from POD 8 (230 K/μL) to POD 13 (70 K/μL).

**Figure 3.**
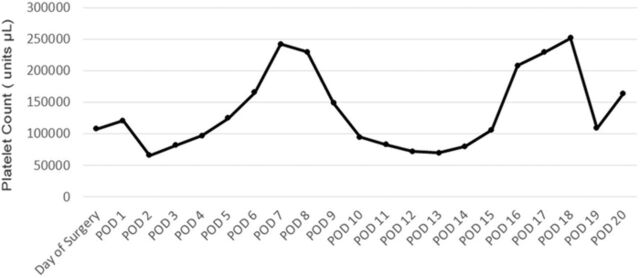
**Biphasic morphology of the platelet count trend suggests heparin-induced thrombocytopenia pathology.** POD, postoperative day.

Studies have shown that a biphasic pattern—an initial rise and then dramatic fall in platelet counts—is strongly correlated to HIT when observed in patients who required <2 hours of CPB and who are 5 days postoperative.^10,18^ Upon review of platelet levels trends, we observed that the patient exhibited a biphasic pattern (Figure 3), suggesting possible signs of HIT pathology.

Diagnosis of HIT in cardiac patients can be difficult. Up to 25% of cardiac surgery patients experience a 40% to 60% decrease in platelet counts within the first 72 hours postoperatively because of blood loss, platelet consumption during CPB, and hemodilution.^8,22-24^ This transient thrombocytopenia can mask early signs of thrombocytopenia attributable to HIT.

Studies have shown that up to 50% of patients undergoing CPB develop anti–platelet factor 4/heparin antibody on POD 5 after treatment with UFH, and on POD 8, 25% of patients still have antibodies, with 15% to 20% having a positive platelet activation assay.^25-27^ However, not everyone who has HIT antibodies develops the disease, and studies report that only 1% to 2% of patients develop clinically significant HIT.^26^ Therefore, caution must be exercised when interpreting laboratory results, and they must be correlated with clinical findings so that patients are not subjected to unnecessary interventions.

Management of HIT involves immediate cessation of heparin and, if postoperative anticoagulation is needed, replacement with direct factor Xa inhibitors (eg, apixaban, rivaroxaban) or direct thrombin inhibitors such as argatroban or bivalirudin, which have been shown to reduce risk of thrombosis by up to 70%.^28^

This case highlights the importance of having a high clinical suspicion for HIT in postoperative cardiac surgery patients. Platelet counts should be monitored closely for all patients who have undergone cardiac surgery, especially during the 5- to 10-day postoperative window. When HIT is suspected, heparin should be discontinued immediately.

As a result of this case, our clinic has implemented strict screenings for HIT and a diagnostic algorithm**.** Consistent with current best practices, we make a presumptive diagnosis of HIT for any patient in the intermediate or high probability risk categories and order anti–platelet factor 4/heparin antibody panel and SRA.

## CONCLUSION

This case illustrates the clinical manifestations of HIT and the life-threatening sequelae in a patient undergoing cardiac surgery. Despite the cessation of heparin and resolution of the patient's thrombocytopenia, the patient did not return to his preoperative condition. This case highlights the importance of having a high clinical suspicion for HIT in all postoperative cardiac surgery patients to allow for early intervention and prevention of severe complications.

## Notes

*This article meets the Accreditation Council for Graduate Medical Education and the American Board of Medical Specialties Maintenance of Certification competencies for Patient Care and Medical Knowledge.*
